# BBTV Nuclear Shuttle Protein Mediates Banana Ubiquitination Pathway Dysregulation

**DOI:** 10.3390/plants15172571

**Published:** 2026-08-24

**Authors:** Xiaoyan Feng, Muhammad Zeeshan Hyder, Rui Meng, Huixiang Yin, Shuli Xian, Jianhua Wang, Yinxue Li, Xuejun Li, Zhixin Liu, Naitong Yu

**Affiliations:** 1 Institute of Tropical Bioscience and Biotechnology, Chinese Academy of Tropical Agricultural Sciences, Haikou 571101, China; fengxiaoyan@catasitbb.cn (X.F.);; 2 Department of Biosciences, COMSATS University Islamabad, Islamabad 45550, Pakistan; zeeshan_hyder@comsats.edu.pk; 3 College of Tropical Crops, Yunnan Agricultural University, Puer 665099, China

**Keywords:** banana bunchy top virus, ssDNA virus, nuclear shuttle protein, ubiquitin–proteasome system, dysregulation

## Abstract

Banana bunchy top virus (BBTV) is a devastating pathogen threatening global banana production. The plant ubiquitin–proteasome system (UPS) governs immune signaling and is frequently subverted by invading viruses, yet the molecular mechanism through which BBTV interferes with host UPS remains unclear. Here, we show that BBTV nuclear shuttle protein (NSP) serves as the core viral effector to disrupt banana ubiquitination homeostasis. RT-qPCR time-series assays confirmed that BBTV infection dynamically remodels the transcription of eight phylogenetically divergent RING-type E3 ubiquitin ligases: four subfamily I E3-SIS3 paralogs and E3-HIP1 are significantly upregulated at 14 dpi and 21 dpi, while E3-BOI and E3-RHA1B are suppressed at 21 dpi. Transient expression screening of all six BBTV-encoded proteins verified that only NSP reproduces the UPS perturbation signature triggered by viral infection. Cross-species sequence alignment identified an evolutionarily conserved FNGSF motif within NSP orthologs of all *Nanoviridae* members. Alanine substitution mutagenesis (NSP^AAAAA^) completely abolished NSP’s capacity to alter E3 ligase transcription. Western blot assays further validated that wild-type NSP induces massive accumulation of ubiquitinated host proteins, whereas the FNGSF-deficient mutant does not disrupt cellular ubiquitination. Phylogenetic analysis revealed that NSP-targeted E3 ligases share low overall sequence similarity but retain conserved catalytic RING domains, indicating that NSP exerts broad-spectrum regulatory effects on host UPS via the FNGSF motif. Collectively, this study reveals a novel pathogenic strategy whereby BBTV NSP recruits diverse host RING E3 ligases via its conserved FNGSF motif to dysregulate plant ubiquitination and elicit plant pathogenicity. Our findings provide two promising targets—the NSP FNGSF motif and defense-associated E3-SIS3 ligases—for developing antiviral agents and breeding BBTV-resistant banana germplasm.

## 1. Introduction

Bananas and plantains are the world’s vital fruit crop, producing over 148 million tons annually across tropical and subtropical regions and supplying food for over 400 million people in more than 140 countries [1]. However, sustainable banana production is severely constrained by multiple viral pathogens including banana bunchy top virus (BBTV; *Babuvirus musae*), banana streak viruses (BSVs; *Badnavirus alphavirgamusae*, *B. deltavirgamusae*, *B. gammavirgamusae*, and *B. iotavirgamusae*), cucumber mosaic virus (CMV; *Cucumovirus CMV*) and banana bract mosaic virus (BBrMV; *Potyvirus musae*) [2,3,4,5]. These viruses cause irreversible growth defects, yield loss and quality deterioration, leading to significant losses to the global banana industry. Notably, banana bunchy top disease (BBTD), triggered by BBTV, has ravaged banana cultivation across tropical Asia and Africa and become the dominant limiting factor for regional banana production [6,7].

BBTV is transmitted exclusively by the banana aphid *Pentalonia nigronervosa,* and infected plants display characteristic stunting, leaf bunching and sterility symptoms [8]. Taxonomically, BBTV belongs to the *Babuvirus* genus within the *Nanoviridae* family, possessing six independent circular single-stranded DNA components (DNA-R, -U3, -S, -M, -C, and -N) ranging from 1.0 to 1.1 kb [9]. Each component is encapsulated into no-envelope icosahedral virions of 17–20 nm in diameter. The DNA-N component encodes the viral nuclear shuttle protein (NSP), which is well-established to mediate nucleocytoplasmic trafficking of viral DNA and facilitate intercellular viral movement [10], yet its virulence functions in manipulating host cellular pathways remain largely uncharacterized.

The ubiquitin–proteasome system (UPS) acts as a core post-translational modification hub governing plant growth, abiotic stress acclimation and multilayered immune signaling. Within this cascade, E3 ubiquitin ligases determine substrate recognition specificity via selective binding and ubiquitination of target proteins, thereby orchestrating pattern-triggered immunity (PTI) and effector-triggered immunity (ETI) at transcriptional and post-translational levels. Conversely, invading pathogens have evolved elaborate co-evolutionary strategies to hijack, repurpose or mimic the host’s E3 ligases to suppress plant defenses, sparking an intensive molecular arms race centered on ubiquitin-mediated protein turnover [11,12]. To date, however, it remains largely unclear how banana E3 ubiquitin ligases respond to BBTV infection, as well as which viral effectors manipulate the host UPS. Mounting evidence reveals that diverse single-stranded DNA (ssDNA) viruses are capable of hijacking the host UPS to facilitate successful infection [13,14]. Nevertheless, there have been no reports showing that BBTV NSP directly modulates the host’s E3 ubiquitin ligases. Additionally, the conserved FNGSF motif of NSP has not yet been identified as the core functional domain responsible for UPS regulation.

Existing commercial banana cultivars nearly lack inherent genetic resistance to BBTV [15]. Deciphering the molecular pathogenic cascade of BBTV-UPS interplay will provide rational targets for engineering virus-resistant banana germplasm. In this work, we dissect the molecular strategy by which BBTV NSP rewires banana ubiquitination pathways, pinpoint the conserved FNGSF motif as the indispensable functional motif for UPS dysregulation, and elaborate the evolutionary divergence of NSP-targeted E3 ligases. Our findings lay a theoretical framework for understanding BBTV–host cellular network interactions and offer promising clues for breeding BBTV-resistant banana varieties via NSP FNGSF motif and E3 ligase-mediated green crop protection.

## 2. Results

### 2.1. Ubiquitination-Related Genes Respond to BBTV Infection

**Figure 1:**
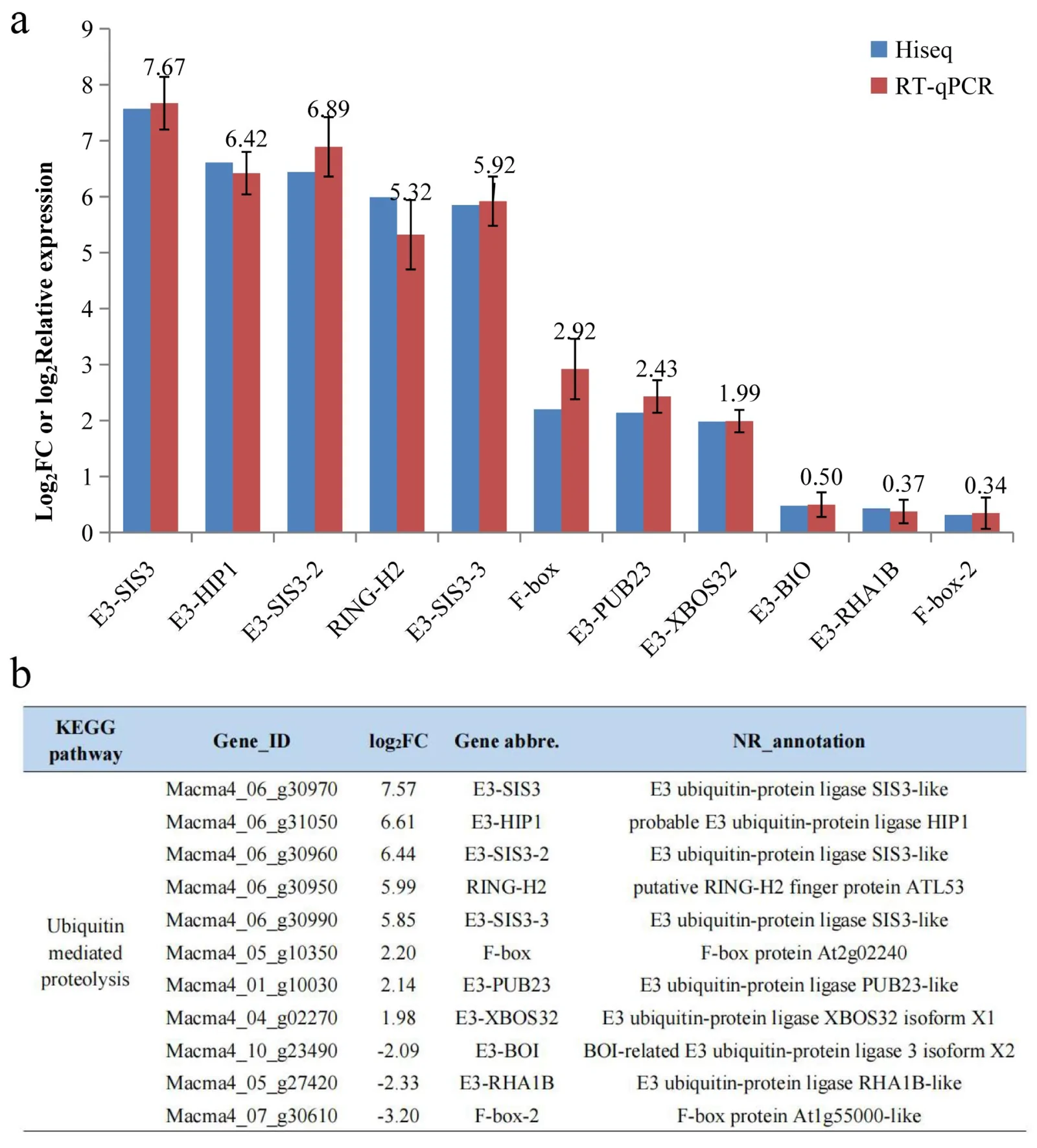
Banana ubiquitination-related genes respond to BBTV infection. (**a**) Comparison of transcription levels of 11 ubiquitination-related genes between Illumina Hiseq 2000 sequencing (blue) and RT-qPCR (red). Error bars represent the standard deviations of three biological replicates. (**b**) The DEGs involved in the ubiquitin-mediated proteolysis pathway are listed here and highlighted in Table S1.

KEGG pathway enrichment analysis identified significant perturbations in UPS-related pathways, including protein processing in the endoplasmic reticulum and ubiquitin-mediated proteolysis (Figure S1), suggesting that BBTV infection disrupts host protein ubiquitination. Gene functional annotation indicated that 11 differentially expressed genes (DEGs) encoding RING-type E3 ubiquitin ligases and F-box proteins were significantly altered in response to BBTV infection. Real-time quantitative PCR (RT-qPCR) validation of these 11 UPS-related DEGs (Table 1) confirmed expression patterns consistent with those derived from transcriptomic data based on log_2_(FC) or log_2_(relative expression) values (Figure 1a).

These DEGs consist of three E3-SIS3s isoforms (E3-SIS3: Macma4_06_g30970; E3-SIS3-2: Macma4_06_g30960; E3-SIS3-3: Macma4_06_g30990), E3-HIP1 (Macma4_06_g31050), RING-H2 (Macma4_06_g30950), two F-box proteins (F-box: Macma4_05_g10350; F-box-2: Macma4_07_g30610), E3-PUB23 (Macma4_01_g10030), E3-XBOS32 (Macma4_04_g02270), E3-BIO (Macma4_10_g23490), and E3-RHA1B (Macma4_05_g27420) (Figure 1b). Collectively, these preliminary results suggested that BBTV infection triggers profound changes in the host protein ubiquitination pathway.

### 2.2. Temporal Expression Patterns of Ubiquitination-Related Genes Under BBTV Infection

**Figure 2:**
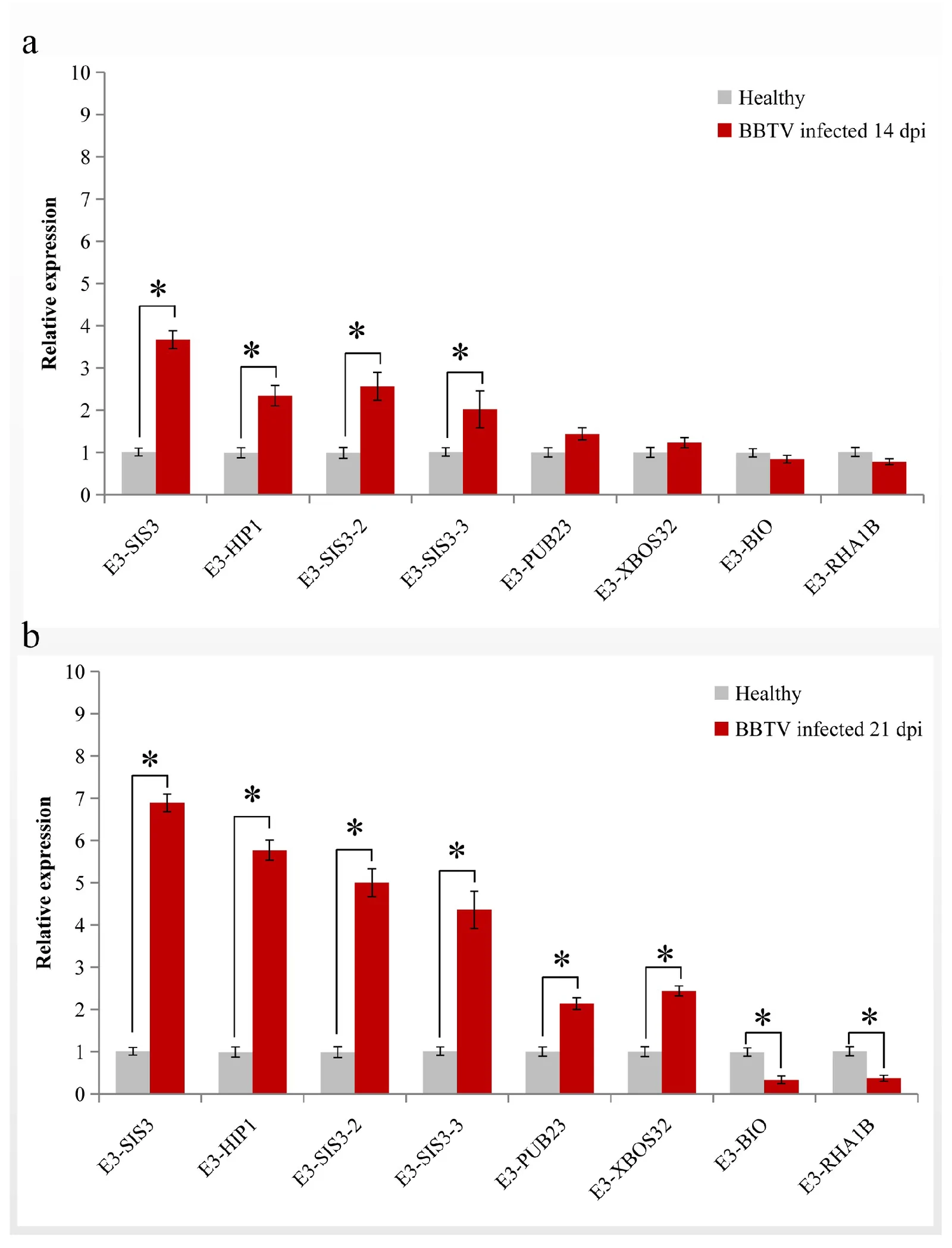
RT-qPCR quantification of transcript levels of E3 ubiquitin ligase genes in healthy banana leaves and BBTV-infected leaves harvested at 14 dpi (**a**) and at 21 dpi (**b**). Error bars represent the standard deviations from three biological replicates. Asterisks “*” indicate statistically significant differences compared with healthy controls (*p* < 0.05).

To verify that BBTV infection disrupts the host UPS, we performed RT-qPCR to quantify transcript abundances of eight E3 ubiquitin ligases in banana leaf tissues sampled at 14 and 21 days post-infection (dpi). The RT-qPCR results revealed distinct temporal expression patterns of these UPS-related genes upon BBTV infection (Figure 2). At 14 dpi, three E3-SIS3 isoforms (E3-SIS3, E3-SIS3-2, and E3-SIS3-3) and E3-HIP1 were significantly upregulated relative to healthy control plants, and their expression levels were further elevated at 21 dpi (Figure 2a,b). In contrast, significant transcriptional changes in E3-PUB23, E3-XBOS32, E3-BIO and E3-RHA1B were only detected at the late-infection stage (21 dpi). Among them, E3-BIO and E3-RHA1B exhibited marked downregulation (Figure 2b). Collectively, the RT-qPCR results further corroborate that BBTV perturbs the host UPS by dynamically regulating the transcription of multiple E3 ubiquitin ligase genes throughout infection.

### 2.3. Banana UPS Dysregulation Is Triggered by BBTV NSP

**Figure 3:**
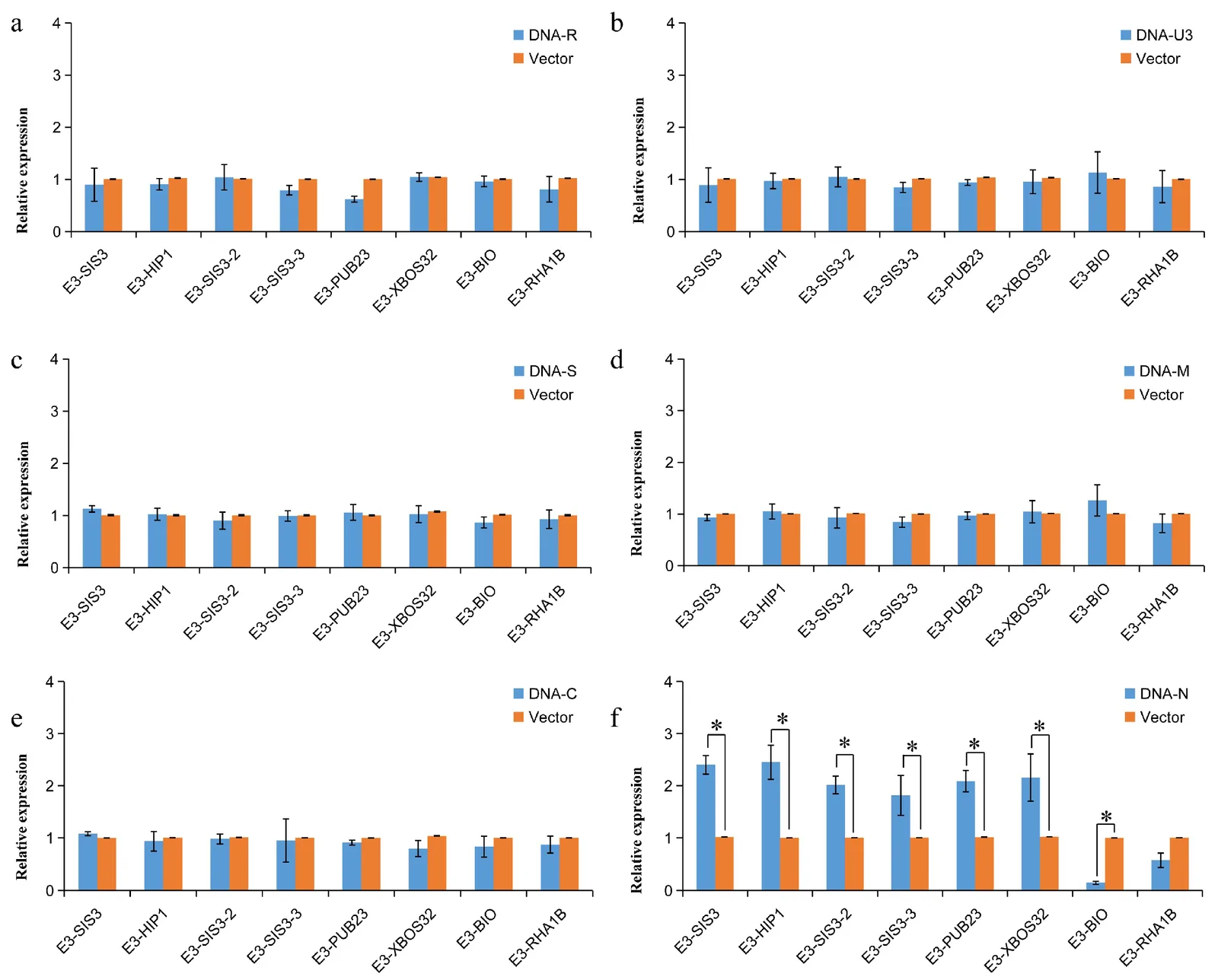
RT-qPCR analysis of transcript abundances of the E3 ubiquitin ligase gene in banana leaves transiently infiltrated with empty pCAMBIA1301 vector (Vector) or constructs expressing individual BBTV proteins. (**a**) DNA-R (Rep), (**b**) DNA-U3 (U3), (**c**) DNA-S (CP), (**d**) DNA-M (MP), (**e**) DNA-C (Clink), and (**f**) DNA-N (NSP). Error bars represent the standard deviations from three biological replicates. Asterisks “*” indicate statistically significant differences (*p* < 0.05).

To identify which viral component of BBTV drives the perturbation of the host protein ubiquitination pathway, we transiently overexpressed each BBTV-encoded protein in banana leaves using the pCAMBIA1301 vector and measured transcript abundances of E3 ubiquitin ligase genes via RT-qPCR. RT-qPCR quantification of eight E3 ubiquitin ligase genes revealed that transient expression of five viral proteins (Rep, U3, CP, MP, and Clink; Figure 3a–e) barely altered the transcription of these target genes. In contrast, NSP overexpression induced profound transcriptional remodeling that recapitulated the E3 ligase expression signature detected upon authentic BBTV infection (Figure 3f). Specifically, NSP significantly upregulated six E3 ligase genes relative to empty vector controls: E3-SIS3 (RE = 2.40), E3-HIP1 (RE = 2.45), E3-SIS3-2 (RE = 2.02), E3-SIS3-3 (RE = 1.82), E3-PUB23 (RE = 2.09), and E3-XBOS32 (RE = 2.16). By contrast, E3-BIO (RE = 0.15) and E3-RHA1B (RE = 0.57) were markedly suppressed upon NSP expression. Together, these results demonstrate that NSP functions as the primary viral effector driving dysregulation of the host UPS.

### 2.4. The Conserved FNGSF Motif of NSP Is Indispensable for UPS Dysregulation

**Figure 4:**
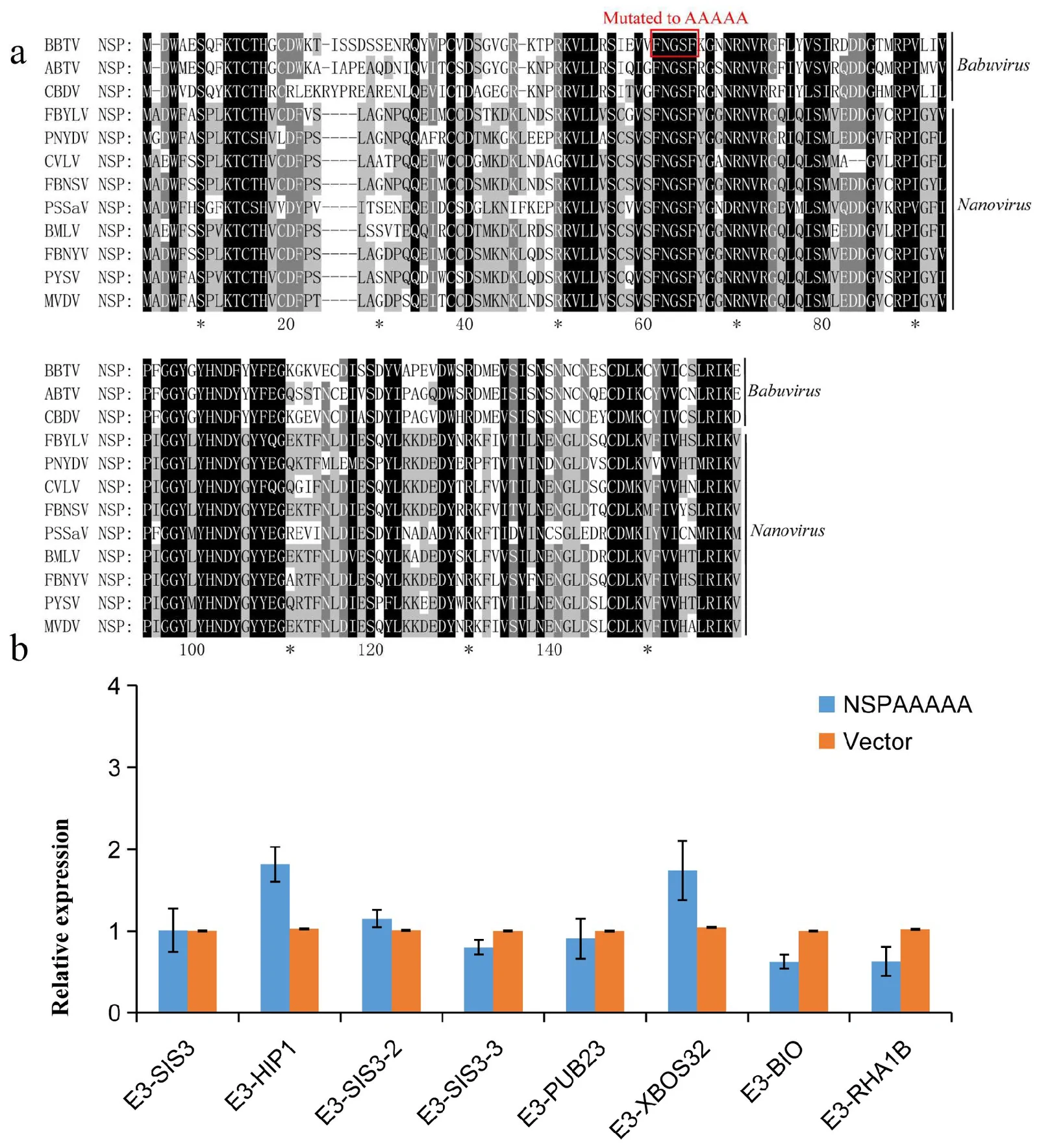
Sequence conservation analysis and functional verification of the NSP FNGSF motif. (**a**) Multiple sequence alignment of NSPs from representative babuviruses and nanovirids; the conserved FNGSF motif and AAAAA substitution mutant are highlighted. Asterisks (*) indicate positions at intervals of 10 amino‑acid residues. (**b**) RT-qPCR analysis of transcript abundances of eight E3 ubiquitin ligase genes in banana leaves infiltrated with the empty pCAMBIA1301 vector (Vector) or the alanine-substituted mutant NSP^AAAAA^. Error bars denote the standard deviations from three independent biological replicates.

The alignment of NSP homologs from diverse nanovirid species revealed a strikingly conserved FNGSF amino acid stretch across all tested viral NSPs (Figure 4a), implying that this motif is evolutionarily conserved and likely carries core biological functions.

To verify whether the FNGSF motif is required for NSP to modulate E3 ubiquitin ligase transcription, pCAMBIA1301-NSP^AAAAA^ was transiently expressed in banana plantlets via agroinfiltration. RT-qPCR quantification of the eight E3 ubiquitin ligase genes showed that expression of NSP^AAAAA^ failed to induce any significant transcriptional shifts in these target genes compared with empty vector controls (Figure 4b). Unlike wild-type NSP, the mutant lost the capacity to upregulate E3-SIS3, E3-HIP1 (non-significant), E3-SIS3-2, E3-SIS3-3, E3-PUB23 and E3-XBOS32 (non-significant), as well as to suppress E3-BIO and E3-RHA1B. Collectively, these mutagenesis results confirm that BBTV NSP hijacks the host UPS in an FNGSF-motif-dependent manner, and this conserved FNGSF sequence is the key functional region governing NSP-mediated dysregulation of host E3 ubiquitin ligases.

### 2.5. The FNGSF Motif Is Required for NSP-Driven UPS Dysregulation by Western Blot Analysis

**Figure 5:**
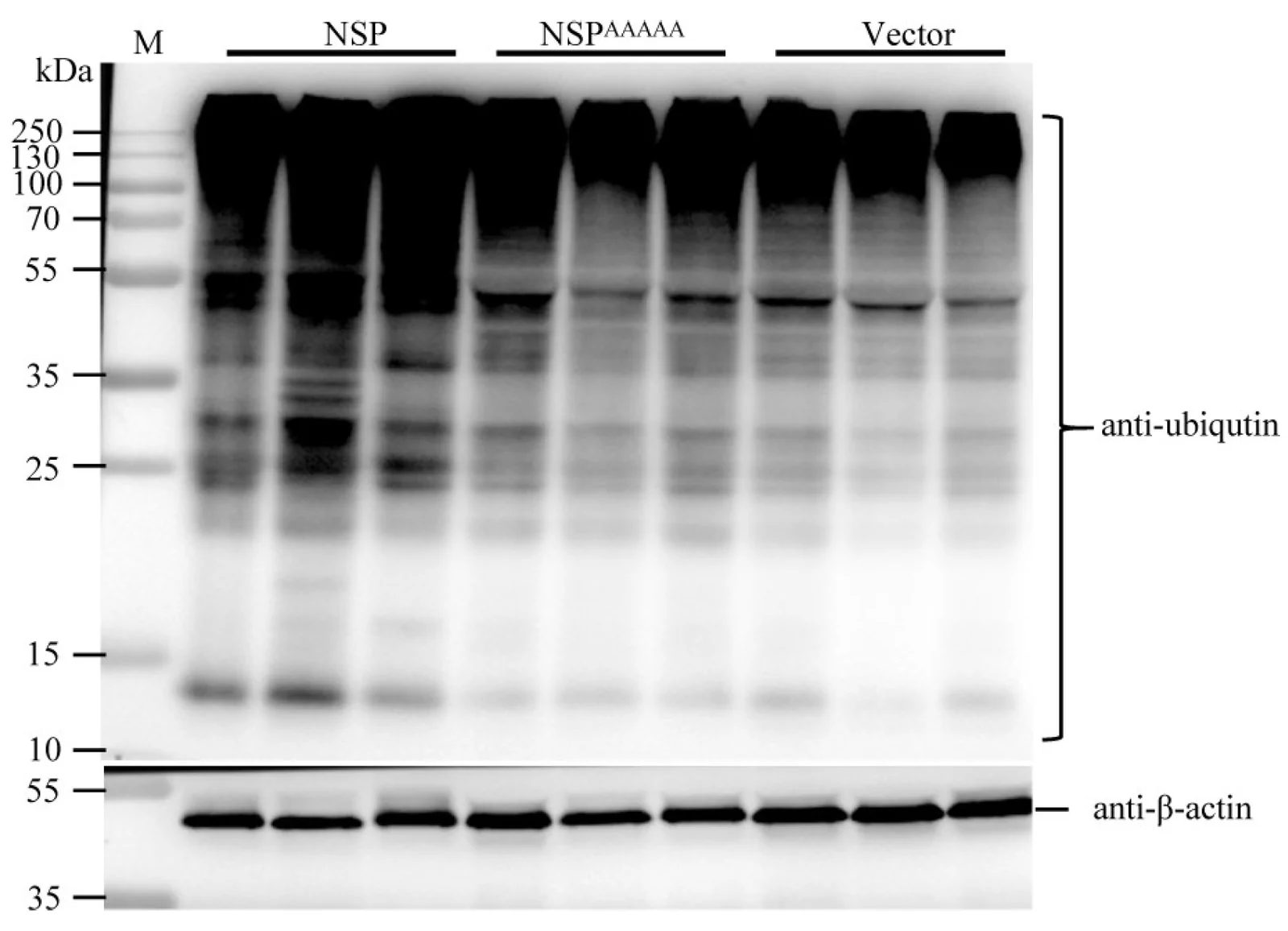
Western blot detection of total ubiquitinated proteins in *N. benthamiana* leaves. Agrobacterium carrying pCAMBIA1301-NSP, pCAMBIA1301-NSP^AAAAA^ or the empty pCAMBIA1301 vector was infiltrated into leaves, and samples were collected at 7 dpi. Immunoblots were probed with anti-ubiquitin antibodies; anti-β-actin was used as a loading control to verify equal protein loading. M: protein molecular-weight marker (10–250 kDa).

To biochemically verify whether the FNGSF motif mediates NSP-induced global ubiquitination, we transiently expressed pCAMBIA1301-NSP, pCAMBIA1301-NSP^AAAAA^, and empty vector control in *N. benthamiana* leaves via agroinfiltration for Western blot detection. The results revealed robust accumulation of high-molecular-weight ubiquitinated proteins (40~250 kDa) in samples expressing wild-type NSP. In contrast, neither NSP^AAAAA^ nor empty vector treatment elevated global ubiquitination levels (Figure 5). This biochemical evidence confirms that the intact FNGSF motif is strictly required for NSP to perturb host UPS activity and boost total cellular ubiquitination.

### 2.6. Phylogenetic Classification, Sequence Identity, and Conserved Domain Architecture of E3 Ubiquitin Ligases

**Figure 6:**
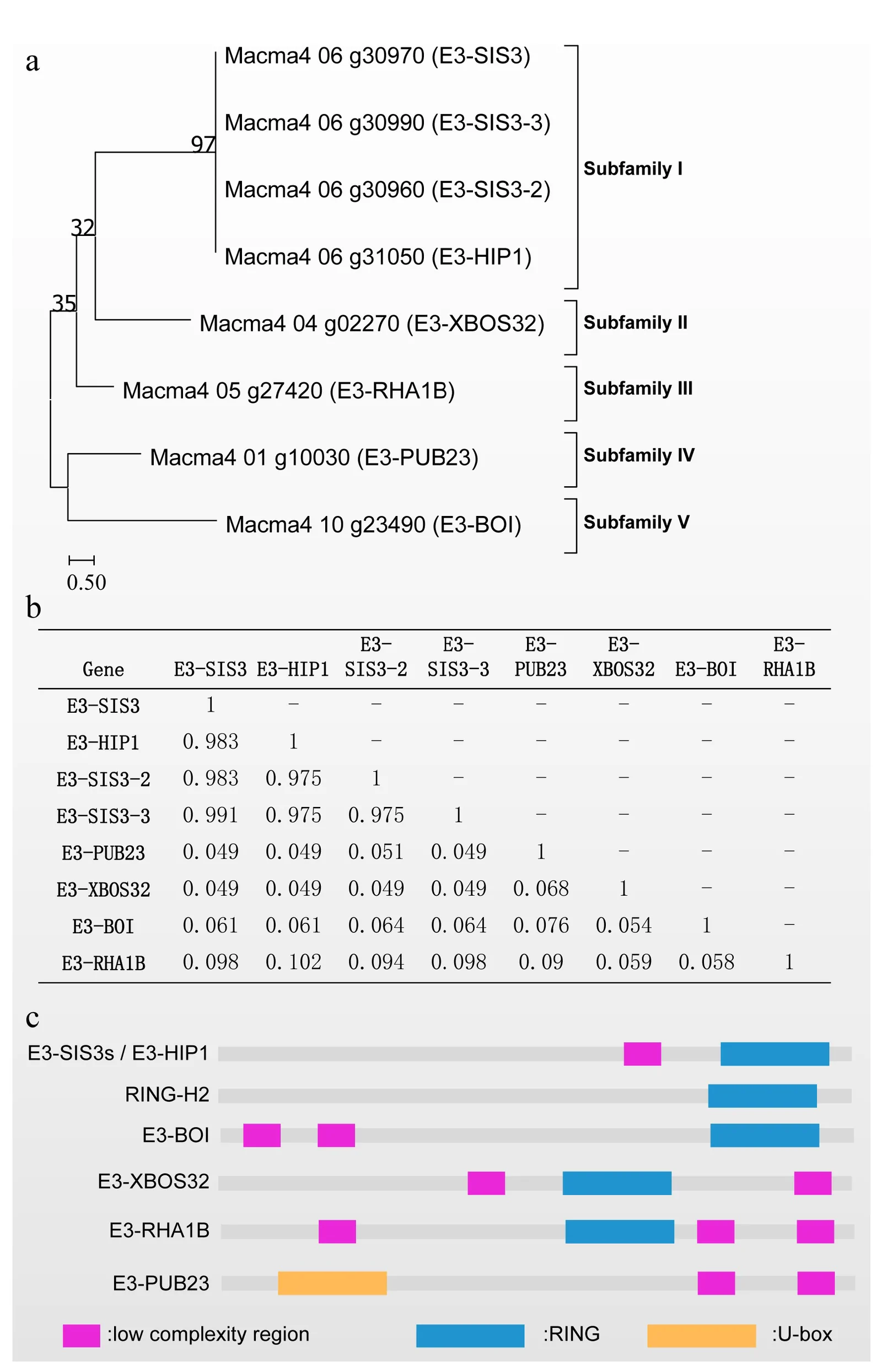
Phylogenetic relationship, pairwise sequence identity matrix and conserved domain architecture of eight E3 ubiquitin ligase proteins. (**a**) A phylogenetic tree was constructed based on the amino acid sequences using the Maximum Likelihood method with the JTT matrix-based model (bootstrap replicates = 1000). Branch labels indicate the percentage of bootstrap support. The analysis included eight sequences, with positions having less than 50% site coverage removed (final dataset: 244 positions). (**b**) Pairwise identity matrix showing sequence similarity percentages between every pair of the eight E3 ligases, calculated from full-length protein alignments. (**c**) Schematic diagram of protein domain architectures predicted via the SMART database. Conserved RING domains and U-box domains are labeled, alongside low-complexity regions. Note: E3-SIS3s represents E3-SIS3, E3-SIS3-2, and E3-SIS3-3.

We performed phylogenetic clustering and pairwise sequence identity analysis on the eight BBTV-responsive E3 ubiquitin ligases identified above. The results showed that these proteins fall into five distinct subfamilies and exhibit extremely low pairwise sequence similarity across most subgroups, with identity values generally below 0.10 (Figure 6a,b). Despite substantial divergence in their full-length amino acid sequences, all eight ligases carry a conserved catalytic RING domain, except E3-PUB23, which contains a U-box domain. Additional auxiliary low-complexity regions are also observed (Figure 6c), consistent with previously reported structural characteristics of plant RING-type E3 ligases [16].

The broad phylogenetic divergence and distinct structural grouping of these eight E3 ligases, along with the consistent transcriptional reprogramming induced by wild-type NSP but lost in the FNGSF-mutated NSP^AAAAA^, collectively suggest that the conserved FNGSF motif of BBTV NSP mediates broad-spectrum perturbation of host ubiquitination signaling via the conserved catalytic RING domain.

## 3. Discussion

This study identified the NSPs of BBTV as the core viral effector that disrupts ubiquitination homeostasis in banana plants. Temporal RT-qPCR results revealed that eight RING-type E3 ubiquitin ligases exhibited differential transcriptional expression patterns during BBTV infection. Among all viral proteins encoded by BBTV, only NSP could recapitulate the dysregulated phenotype of the host UPS. The conserved FNGSF motif, widely distributed across *Nanoviridae*, acts as a critical functional site for NSP to remodel the expression of host E3 ligases and promote host protein ubiquitination. Alanine substitution of this motif completely abolished the above regulatory functions of NSP. Despite substantial evolutionary divergence among these eight E3 ligases, all except E3-PUB23 (to be experimentally validated) retain the conserved RING catalytic domain, enabling NSP to broadly interfere with the host ubiquitin regulatory network via the FNGSF motif. In summary, this study uncovered an unreported viral pathogenic mechanism: BBTV NSP targets and modulates multiple RING-type E3 ubiquitin ligases through the FNGSF motif, thereby eliciting pathogenicity in host plants.

It should be clarified that the selection of 14 dpi and 21 dpi for temporal expression profiling was based on the infection and symptom development characteristics of BBTV. BBTV possesses a long latent period; no significant expression changes were detected for host E3 ubiquitin ligase genes at early infection stages (5 dpi, 7 dpi, and 10 dpi). Distinct differential expression of E3 ligase genes first emerged at 14 dpi and became more pronounced at 21 dpi. Therefore, these two key time points were chosen to systematically characterize the temporal expression dynamics of banana E3 ligases upon BBTV infection. In addition, viral symptoms generally develop approximately one month after BBTV inoculation [17], which further rationalizes the choice of these sampling time points.

Transient expression screening of six BBTV-encoded proteins demonstrated that only NSP induced global transcriptional reprogramming of ubiquitin system-related E3 ubiquitin ligases in banana, and this expression pattern was highly consistent with transcriptomic profiles of banana tissues naturally infected with BBTV [18]. Multiple sequence alignment of representative homologous NSPs from the family *Nanoviridae* identified a highly conserved FNGSF amino acid motif. Polar and aromatic residues within this motif are classic functional residues mediating protein–protein interactions, nuclear localization signals (NLSs), and nuclear export signals (NESs), implying that the FNGSF motif may mediate physical binding between NSP and host ubiquitin regulatory components, facilitating viral nucleocytoplasmic trafficking and suppression of host immunity [19,20]. Full alanine substitution of the FNGSF motif completely eliminated the capacity of NSP to remodel E3 ligase transcription, confirming that an intact FNGSF sequence is an indispensable functional motif for NSP to perturb the host ubiquitin system. Glycine within the FNGSF motif lacks a side chain, which confers high flexibility to the peptide chain and enables it to mimic the spatial conformation of the C-terminal Gly-Gly (GG) motif of ubiquitin. This motif is misrecognized by host E3 ubiquitin ligases, which not only blocks the normal transfer of ubiquitin to target proteins but also triggers futile ubiquitination reactions that deplete cellular free ubiquitin and E2 enzymes, ultimately disrupting the host ubiquitination pathway [21]. Biochemical assays were performed using *N. benthamiana* in this study. The results were consistent with the transcriptional data, further verifying the mechanism by which NSP induces dysfunction of the UPS at the protein level.

Notably, our initial attempts to directly detect ubiquitination fluctuations in *Agrobacterium*-infiltrated banana leaves yielded faint Western blot signals and unreliable results, stemming from technical limitations including low transient transformation efficiency of banana epidermal cells and limited *Agrobacterium* infiltration area. To address this bottleneck, we adopted *N. benthamiana*, a model plant with extremely high transient transformation efficiency, for biochemical validation. Protein detection results fully matched transcriptional data: wild-type NSP drastically increased global ubiquitinated protein levels *in planta*, while the FNGSF deletion mutant lost this regulatory activity, further verifying that the FNGSF motif serves as the core functional site through which NSP disrupts host ubiquitin homeostasis.

Temporal RT-qPCR data demonstrated dynamic, infection-stage-specific expression regulation of banana RING-type E3 ligases during BBTV infection. Four subfamily I RING E3 genes (*E3-SIS3*, *E3-SIS3-2*, *E3-SIS3-3*, and *E3-HIP1*) were markedly upregulated at 14 dpi, with expression levels further elevated at 21 dpi. In contrast, transcriptional repression of *E3-BOI* and *E3-RHA1B* was only observed at 21 dpi. Sequence similarity analysis showed that these four early-induced E3-SIS3 paralogs share over 98% amino acid identity and cluster within a single evolutionary clade. Nevertheless, evolutionary analysis of subfamily I-V NSP-responsive E3 ligases revealed extreme genetic divergence, with most sharing less than 10% sequence identity; even so, all of these proteins harbor the signature RING catalytic core domain characteristic of plant ubiquitin ligases [22]. The broad targeting capacity of NSP toward evolutionarily divergent E3 ligases indicates that its conserved FNGSF motif acts as a universal pathogenic module governing host ubiquitin signaling networks, which rely on the RING catalytic core domain.

E3 ubiquitin ligases are categorized into three major subfamilies based on catalytic domain architecture: RING, U-box, and HECT. Among them, RING-type E3s function as central regulators of plant stress responses and immune defense [23,24]. RING-type E3s positively modulate eukaryotic stress responses by mediating substrate ubiquitination and proteasomal degradation [25], consistent with our finding that BBTV NSP strongly induces the upregulation of six *E3* genes at early infection stages. Functional research on homologous E3 ligases in other plant–pathogen interaction systems provides cross-species mechanistic references for this study. The kiwifruit U-box E3 protein PUB23 negatively regulates bacterial disease resistance by degrading transcription factor GT1; silencing *PUB23* significantly enhances pathogen-triggered ROS bursts and activation of defense genes [26].

In addition, downregulation of *E3-BOI* and *E3-RHA1B* at late-infection stages constitutes a secondary immune suppression strategy deployed by BBTV. Functional studies of *Arabidopsis* homologs have confirmed that BOI and RHA1B mediate ubiquitination and degradation of CC-NBS-LRR immune receptors, negatively regulating NLR-triggered cell death and effector-triggered immunity (ETI) [27,28]. By repressing these two immunity-suppressive E3 genes, BBTV balances host hypersensitive cell death and systemic viral spread, generating a favorable intracellular microenvironment for sustained viral proliferation. Collectively, these studies illustrate that pathogen targeting of distinct host E3 ligases to fine-tune plant immunity represents a conserved co-evolutionary strategy, and BBTV NSP executes this classic pathogenic mechanism via its conserved FNGSF motif.

Previous model plant studies on nanoviruses and geminiviruses provide molecular evidence supporting our conclusions. Krapp et al. demonstrated that the NSP of Pea necrotic yellow dwarf virus (PNYDV; *Nanovirus necropisi*, family *Nanoviridae*) carries a conserved FNGSF motif, whereas the C-terminus of Abutilon mosaic virus (AbMV; *Begomovirus bauri*, family *Geminiviridae*) NSP contains a homologous FVSF motif. NSPs from both viral families rely on these conserved motifs to bind *Arabidopsis* G3BP homologs, sequester host stress granule (SG) components, and block SG-dependent basal antiviral defense pathways. Alanine substitution of key phenylalanine residues within the FNGSF/FVSF motifs completely abolished physical interactions between viral NSPs and AtG3BP, eliminating their ability to perturb host stress responses [19]. This phenotype highly aligns with our observation that FNGSF mutagenesis abolishes the ubiquitin-regulatory functions of BBTV NSP. This cross-family conserved mechanism indicates that *Nanoviridae* NSPs utilize conserved aromatic amino acid motifs to capture core host regulatory proteins and attenuate basal plant resistance as a universal evolutionary strategy. Accordingly, this phenylalanine may play a critical structural role in NSP-induced dysfunction of the UPS. Moreover, it represents an additional core site in the molecular model whereby plant DNA viruses antagonize host immunity through short conserved peptide motifs.

F-box proteins serve as substrate recognition subunits of the SCF-E3 complex and possess no E3 catalytic activity. The expression of F-box proteins and their isoforms is markedly upregulated during viral infection, whereas no significant changes are observed when NSP is expressed alone. Previous studies have demonstrated that the Clink protein of nanoviruses specifically binds to SKP1 to regulate the F-box-associated pathway [29]. Accordingly, transcriptional fluctuations of F-box family genes are more likely induced by BBTV Clink rather than NSP. For this reason, the interaction between Clink and the SCF-E3 complex was not investigated in the present study, and it will be explored in future research. Although Macma4_06_g30950 is annotated as a putative RING-H2 protein containing a RING domain, its expression is completely unregulated in the NSP transient expression system. Differential expression of this RING-H2 gene was only identified in the global screening upon viral infection, so it was excluded from subsequent functional verification. We hypothesize that this protein may also act as a substrate recognition subunit of the SCF-E3 complex.

While this study preliminarily elucidated the pathogenic mechanism of BBTV NSP, several critical questions remain to be addressed in future research. First, yeast two-hybrid, co-immunoprecipitation, and bimolecular fluorescence complementation (BiFC) assays are required to verify direct physical interactions between NSP and banana E3 ligases, and to clarify whether NSP functions as a substrate or adaptor protein during E3-mediated ubiquitination reactions. Second, ubiquitin proteomics can be applied to identify host target proteins of NSP-E3 complexes, enabling precise dissection of the core immune pathways suppressed by BBTV. Third, banana transgenic overexpression and CRISPR-Cas9 knockout systems will be used to validate the in vivo functions of core E3 genes (*E3-SIS3s* and *E3-RHA1B*) in resistance to BBTV infection. From an applied translational perspective, the conserved FNGSF motif of NSP represents an ideal antiviral target. Genetic editing to disrupt the interaction interface between NSP and host E3 proteins can facilitate the breeding of banana germplasm resistant to bunchy top disease and support the eco-friendly sustainable production of tropical crops. In conclusion, the FNGSF motif of NSP and E3-SIS3 ligases serves as a key target for antiviral drug development and disease resistance breeding in banana.

## 4. Materials and Methods

### 4.1. Gene Ontology (GO) and KEGG Pathway Enrichment Analyses of UPS-Related DEGs

To elucidate the molecular pathogenic mechanisms triggered by BBTV infection, transcriptome data deposited under NCBI accession number SRP129855 [18] were re-analyzed using the BMKCloud online analysis platform (Biomarker Technologies, Beijing, China). Genes with significant expression changes were screened with thresholds of (|log_2_(FC)| ≥ 1.7 and a −log_10_(FDR) ≥ 2.0). In total, 147 DEGs were filtered from 38,643 unigenes, including 63 upregulated and 84 downregulated transcripts (Table S1 and Figure S2).

For GO enrichment, the R package GOseq was applied to classify the biological functions of the identified DEGs. Briefly, functional GO annotations were retrieved by Blast2GO based on the top BLASTx alignments against the NCBI non-redundant (nr) protein database [30]. GO terms with *p* < 0.05 were defined as significantly enriched. For KEGG pathway enrichment, pathway annotation and enrichment statistics of DEGs were performed with KOBAS software (v3.0) referencing the public KEGG genome database. Pathways with *p* < 0.05 were regarded as significantly enriched functional pathways [31].

### 4.2. RT-qPCR Validation of UPS-Related Gene Expression upon BBTV Infection

To verify the differential expression of UPS-associated genes revealed by transcriptome profiling, 11 UPS-related candidate genes were selected for RT-qPCR quantification to compare transcript abundances between BBTV-infected and healthy banana leaf samples. All gene-specific primer sequences used in this study are listed in Table 1. Quantitative amplification was performed on a StepOne Real-Time PCR System (Applied Biosystems, Foster City, California, USA) with the following thermal cycling program: an initial denaturation step at 95 °C for 5 min, followed by 40 cycles of 95 °C for 15 s and 60 °C for 60 s. The housekeeping gene glyceraldehyde 3-phosphate dehydrogenase (GAPDH) was employed as an internal reference for normalization of relative transcript levels, which were calculated based on log_2_(FC) or log_2_(relative expression) values.

For subsequent temporal expression detection, we further quantified the expression of eight E3 ubiquitin ligase genes (E3-SIS3, E3-SIS3-2, E3-SIS3-3, E3-HIP1, E3-PUB23, E3-XBOS32, E3-BIO and E3-RHA1B) in banana leaf tissues harvested at 14 and 21 dpi. Total RNA extracted from the diseased sample was reverse-transcribed to cDNA using the HiFiScript cDNA Synthesis Kit (CWBIO Biotech, Beijing, China). The RT-qPCR reaction setup and thermal cycling parameters remained identical to those described above, while expression levels were calculated using the 2^−ΔΔCt^ method.

### 4.3. Verification of Host UPS Dysregulation Induced by BBTV NSP in Host

To screen viral modulators targeting the UPS, each coding gene of BBTV was cloned into the pCAMBIA1301 vector and transiently expressed in banana plantlets via *Agrobacterium tumefaciens* strain GV3101-mediated transformation. The transient expression assay of recombinant plasmids in the *Musa* AAA Cavendish subgroup cv. Brazil banana was performed following a previously described protocol [32].

At 72 h post-inoculation (hpi), total RNA was extracted and reverse-transcribed into first-strand cDNA in accordance with the aforementioned methods. RT-qPCR was subsequently conducted to quantify the transcript levels of the eight ubiquitination-related genes in banana plantlets expressing individual BBTV proteins, with plantlets transformed with the empty pCAMBIA1301 vector serving as the control (Table 1). All RT-qPCR amplifications were carried out using the thermal cycling program established above.

### 4.4. Identification and Functional Analysis of NSP FNGSF Motif in UPS Dysregulation

To identify the functional motif responsible for NSP-triggered perturbation of the host UPS, we first performed multiple sequence alignment of NSP homologs from 12 diverse nanovirid species, including both babuviruses and nanoviruses. The alignment was conducted using Clustal X 1.83 (https://bio.tools/clustalw) and edited with GeneDoc software (https://www.softpedia.com/get/Science-CAD/GeneDoc.shtml). The NSP sequences used in this alignment were retrieved from the NCBI GenBank database (https://www.nlm.nih.gov/oet/ed/navigator/nucleotide/index.html), with the following accession numbers: BBTV NSP (AZL93962.1), abaca bunchy top virus (ABTV; *Babuvirus abacae*) NSP (YP_001661656.1), cardamom bushy dwarf virus (CBDV; *Babuvirus cardamomi*) NSP (AGG38930.1), faba bean yellow leaf virus (FBYLV; *Nanovirus flaviviciae*) NSP (CCF74117.1), pea necrotic yellow dwarf virus (PNYDV; *Nanovirus necropisi*) NSP (AHC72275.1), cow vetch latent virus (CVLV; *Nanovirus viciacraccae*) NSP (ATY70081.1), faba bean necrotic stunt virus (FBNSV; *Nanovirus necropumiliviciae*) NSP (ACX50516.1), parsley severe stunt-associated virus (PSSaV; *Nanovirus petroselini*) NSP (QIH29490.1), black medic leaf roll virus (BMLV; *Nanovirus medicagonis*) NSP (YP_008997799.1), faba bean necrotic yellows virus (FBNYV; *Nanovirus necroflaviviciae*) NSP (NP_619573.1), pea yellow stunt virus (PYSV; *Nanovirus flavipisi*) NSP (YP_008997808.1) and milk vetch dwarf virus (MVDV; *Nanovirus astragali*) NSP (NP_619764.1).

To verify whether the FNGSF motif is required for NSP to modulate the transcription of E3 ubiquitin ligase genes, an alanine substitution mutant NSP^AAAAA^ was synthesized by Anhui General Biotechnology Co. (Chuzhou City, Anhui, China). The synthetic fragment was then cloned into the pCAMBIA1301 vector using the *Spe*I and *Kpn*I restriction sites. Transient expression of this mutant in banana plantlets via agroinfiltration was performed as described in the previous section [32]. Total RNA extractions and RT-qPCR amplifications were carried out as established above.

### 4.5. Western Blot Analysis of the FNGSF Motif Is Required for NSP-Driven UPS Dysregulation

To investigate whether the FNGSF motif is essential for NSP-induced UPS dysregulation at the protein level, three constructs including pCAMBIA1301-NSP, pCAMBIA1301-NSP^AAAAA^ and empty pCAMBIA1301 were individually transformed into *Agrobacterium* and infiltrated into *N. benthamiana* leaves. Leaf samples were collected at 7 dpi and used for total protein isolation. The protein samples were then subjected to a Western blot to analyze the expression levels of total ubiquitinated proteins. The primary antibody anti-ubiquitin (PTM Biolabs, PTM-1107, Hangzhou, China) was applied at 1:1000, and the secondary antibody HRP-conjugated goat anti-rabbit IgG (Beyotime, A0208, Shanghai, China) was used at a dilution of 1:2000. Anti-β-actin antibody was used as a loading control to ensure equal protein loading across samples. Western blot analysis was performed according to the protocol described in the reference [33].

### 4.6. Phylogenetic Classification, Sequence Identity, and Conserved Domain Architecture of Eight E3 Ubiquitin Ligases

A phylogenetic tree was constructed based on amino acid sequences using the Maximum Likelihood algorithm under the JTT matrix-based substitution model. Bootstrap support values generated from 1000 replicate iterations are shown at each branch node, with branch annotations representing the percentage of bootstrap confidence. A total of eight protein sequences were incorporated into the phylogenetic dataset; sites with less than 50% coverage across all sequences were eliminated, yielding a final alignment of 244 amino acid positions. All phylogenetic analyses were executed in MEGA 12 (v12.1.2) [34]. Pairwise sequence identity percentages among the eight E3 ubiquitin ligases were generated based on full-length protein sequence alignments, which were processed using BioEdit v7.0.9.0 (https://thalljiscience.github.io/). Schematic diagrams of protein domain architectures were predicted via the SMART online resource (https://smart.embl.de/smart/change_mode.cgi). Conserved RING domains and U-box domains were annotated, along with low-complexity regions.

## 5. Conclusions

This study demonstrates that BBTV infection dynamically remodels the banana UPS by modulating the transcription of phylogenetically distinct RING-type E3 ubiquitin ligases. Specifically, four subfamily I E3-SIS3 paralogs and E3-HIP1 are significantly upregulated at the early infection stage, whereas E3-BOI and E3-RHA1B are suppressed in late infection, indicative of stage-specific reprogramming of host ubiquitin signaling during BBTV pathogenesis. Among all six BBTV-encoded proteins, the NSP is identified as the core virulence effector responsible for UPS dysregulation in banana. Alone, transient NSP expression recapitulates the perturbed transcriptional profile of E3 ligases in virus-infected plants, while other viral proteins exert minimal impacts on UPS-related gene expression.

A highly conserved FNGSF amino acid motif was identified across nanovirid NSP orthologs via cross-species sequence alignment. Alanine substitution mutagenesis of this motif (NSP^AAAAA^) completely abrogates NSP’s ability to alter E3 ligase transcription and enhance global cellular ubiquitination. Western blot assays in *N. benthamiana* further validate these findings at the protein level, verifying the FNGSF sequence as an essential functional domain for NSP to hijack the host UPS.

Phylogenetic analysis shows that NSP-targeted E3 ligases share low overall sequence homology but possess conserved catalytic RING domains. Collectively, these results indicate that BBTV NSP mediates broad-spectrum modulation of host ubiquitination dependent on its conserved FNGSF motif, rather than targeting individual host E3 ubiquitin ligases.

## Figures and Tables

**Table 1:** Complete list of oligonucleotide primers used in this study.

Primer Name	Primer Sequence (5′-3′)	Targeted Gene
E3-SIS3-qF	GCTCCGGTGGTGATCAAACTC	*E3-SIS3*
E3-SIS3-qR	AGCCGTCTCGACTAGATCATTG	
E3-HIP1-qF	GCCCCGGTGGTGATCAAACTC	*E3-HIP1*
E3-HIP1-qR	AACCGTCTCGACTAGATCATTGC	
E3-SIS3-2-qF	GCCCCGGTGGTGATCAAACTC	*E3-SIS3-2*
E3-SIS3-2-qR	AGCCATCTCGACTGGATCATTGC	
RING-H2-qF	CCACACCTTTCTCTTCACTCTC	*RING-H2*
RING-H2-qR	ATGTCATAACCATAGACGCCG	
E3-SIS3-3-qF	ATCGAAGGTCGAGTCGTGGAG	*E3-SIS3-3*
E3-SIS3-3-qR	CAGCTCCTTCACCGCGTCCGT	
F-box-qF	GGTTCCTGCCGTCCAATATC	*F-box*
F-box-qR	CCGCCCCAAGTAATCCATAG	
E3-PUB23-qF	ATCTGACACTCACCCCAAAC	*E3-PUB23*
E3-PUB23-qR	ATCGTATTTAGAGCCTTCGCC	
E3-XBOS32-qF	TCCCCATACTTATCTCTTCCCC	*E3-XBOS32*
E3-XBOS32-qR	ACACAGGTAAAGGGCACATC	
E3-BOI-qF	GTTAGCCATGGAAGTGAGTACG	*E3-BOI*
E3-BOI-qR	TGTAAATCAGTGTCCCCGTTG	
E3-RHA1B-qF	ACTGCGACTTGAGACTTGTG	*E3-RHA1B*
E3-RHA1B-qR	ACTCTTCCTGCTCTCCTACC	
F-box-2-qF	TGGAACTGCAGAATTCTACCTG	*F-box* *-* *2*
F-box-2-qR	GGTCTCATGGTCTGTTGCTC	
GAPDH-qF	GACAGATTTGGAATAGTGGAGGGTTTG	*GAPDH*
GAPDH-qR	CCAGTGCTGCTAGGAATGATGTTGAAG	
NSP-gF	GGACTAGTATGGATTGGGCAGAATCAC	p1300-NSP, p1300-NSP^AAAAA^
NSP-gR	CGGGATCCTTCCTTTATTCTTAACGAAC	

## Data Availability

The original contributions presented in this study are included in the article/Supplementary Materials. Further inquiries can be directed to the corresponding authors.

## Supplementary Materials

The following supporting information can be downloaded at https://www.mdpi.com/article/10.3390/plants15172571/s1: Table S1: List of the differentially expressed genes (DEGs) between BBTV-infected and healthy banana leaves; Figure S1: KEGG enrichment analysis of differentially expressed genes between BBTV-infected and healthy banana leaves; Figure S2: Transcriptomic profiling reveals differentially expressed genes (DEGs) between BBTV-infected and healthy banana leaves.

## References

[1] Tripathi L., Ntui V.O., Tripathi J.N. (2020). CRISPR/Cas9-based genome editing of banana for disease resistance. Curr. Opin. Plant Biol..

[2] Mapinda J.J., Hugo A.K., Shinzeh J.K., Edward S. (2025). Modelling the effects of virus-resistant planting material on the transmission dynamics of banana bunchy top disease. Sci. Rep..

[3] Ashraf M.A., Ali B., Fareed M., Sardar A., Saeed E., Islam S., Bano S., Yu N.T. (2025). In silico identification of banana high-confidence microRNA binding sites targeting banana streak GF virus. Appl. Microbiol..

[4] Srivastava N., Kapoor R., Kumar R., Kumar S., Saritha R.K., Kumar S., Baranwal V.K. (2019). Rapid diagnosis of cucumber mosaic virus in banana plants using a fluorescence-based real-time isothermal reverse transcription-recombinase polymerase amplification assay. J. Virol. Methods.

[5] Kovileri M.M., Nair S., Loius V. (2023). One-step reverse transcription-loop mediated isothermal amplification (RT-LAMP) for closed-tube colorimetric detection of banana bract mosaic virus in banana (*Musa* spp.). 3 Biotech.

[6] Masangwa J.I.G., Pareta N.F., Moses P., Hřibová E., Doležel J., Fandika I., Massart S. (2026). Surveillance and molecular characterization of banana viruses associated with *Musa* germplasm in Malawi. PLoS ONE.

[7] Mendoza N.A.P., Mendoza J.S., Thomas J.E., Dela Cueva F.M. (2024). Alternative hosts of banana bunchy top virus in the Philippines and the first evidence of seed transmission of BBTV. Front. Plant Sci..

[8] Ogwal G., Wasswa P., Ocimati W., Omondi B.A., Tazuba A.F., Otim M.H., Blomme G. (2025). Screening for the efficacy of botanicals and soaps in controlling the banana aphid *Pentalonia nigronervosa* (Hemiptera: Aphididae) under laboratory and screenhouse conditions. Insects.

[9] Yu N.T., Zhang Y.L., Feng T.C., Wang J.H., Kulye M., Yang W.J., Lin Z.S., Xiong Z., Liu Z.X. (2012). Cloning and sequence analysis of two banana bunchy top virus genomes in Hainan. Virus Genes.

[10] Ji X.L., Yu N.T., Qu L., Li B.B., Liu Z.X. (2019). Banana bunchy top virus (BBTV) nuclear shuttle protein interacts and re-distributes BBTV coat protein in *Nicotiana benthamiana*. 3 Biotech.

[11] Wu Y., Tao Z., Qin Z., Li Y., Zhao M., Zhao S., Wu J. (2026). E3 ubiquitin ligases in plant defense: Mechanisms and prospects for resistance breeding. Sci. China Life Sci..

[12] Hamada N., Hand K.A., Shabek N. (2026). Ubiquitin ligases in plant immunity: Structural mechanisms and signaling. Plant J..

[13] Chen Z.Q., Zhao J.H., Chen Q., Zhang Z.H., Li J., Guo Z.X., Xie Q., Ding S.W., Guo H.S. (2020). DNA geminivirus infection induces an imprinted E3 ligase gene to epigenetically activate viral gene transcription. Plant Cell.

[14] Lai J., Chen H., Teng K., Zhao Q., Zhang Z., Li Y., Liang L., Xia R., Wu Y., Guo H. (2009). RKP, a RING finger E3 ligase induced by BSCTV C4 protein, affects geminivirus infection by regulation of the plant cell cycle. Plant J..

[15] Drenth A., Kema G.H.J. (2021). The vulnerability of bananas to globally emerging disease threats. Phytopathology.

[16] Liu R., Xia R., Xie Q., Wu Y. (2021). Endoplasmic reticulum-related E3 ubiquitin ligases: Key regulators of plant growth and stress responses. Plant Commun..

[17] Chabi M., Dassou A.G., Adoukonou-Sagbadja H., Thomas J., Omondi A.B. (2023). Variation in symptom development and infectivity of banana bunchy top disease among four cultivars of *Musa* sp. Crops.

[18] Yu N.T., Xie H.M., Zhang Y.L., Wang J.H., Xiong Z., Liu Z.X. (2019). Independent modulation of individual genomic component transcription and a cis-acting element related to high transcriptional activity in a multipartite DNA virus. BMC Genom..

[19] Krapp S., Greiner E., Amin B., Sonnewald U., Krenz B. (2017). The stress granule component G3BP is a novel interaction partner for the nuclear shuttle proteins of the nanovirus pea necrotic yellow dwarf virus and geminivirus abutilon mosaic virus. Virus Res..

[20] Kosugi S., Hasebe M., Matsumura N., Takashima H., Miyamoto-Sato E., Tomita M., Yanagawa H. (2009). Six classes of nuclear localization signals specific to different binding grooves of importin alpha. J. Biol. Chem..

[21] Glassman C.R., Baek K., Hou G., Zeng Q., Nardone C., Juergens K.B., Fujimura E., O’Leary C.N., Li M.Z., Paulo J.A. (2026). Virome-wide ubiquitin ligase discovery reveals diverse mechanisms of immune evasion. Science.

[22] Kong X., Li F., Shang J., Ji Y., Li Y., Hussain S., Zhang X., Ahmed Z.F.R. (2025). Genome-wide analysis of the RING-H2 E3 ubiquitin ligase SlATL family in tomato. Sci. Rep..

[23] Stone S.L. (2014). The role of ubiquitin and the 26S proteasome in plant abiotic stress signaling. Front. Plant Sci..

[24] Al-Saharin R., Hellmann H., Mooney S. (2022). Plant E3 ligases and their role in abiotic stress response. Cells.

[25] Zheng N., Shabek N. (2017). Ubiquitin ligases: Structure, function, and regulation. Annu. Rev. Biochem..

[26] Wang T., Wang G., Zhang J., Xuan J. (2024). E3 ubiquitin ligase PUB23 in kiwifruit interacts with trihelix transcription factor GT1 and negatively regulates immune responses against *Pseudomonas syringae* pv. *actinidiae*. Int. J. Mol. Sci..

[27] Huang J., Wu X., Gao Z. (2021). The RING-type protein BOI negatively regulates the protein level of a CC-NBS-LRR in *Arabidopsis*. Biochem. Biophys. Res. Commun..

[28] Kud J., Wang W., Gross R., Fan Y., Huang L., Yuan Y., Gray A., Duarte A., Kuhl J.C., Caplan A. (2019). The potato cyst nematode effector RHA1B is a ubiquitin ligase and uses two distinct mechanisms to suppress plant immune signaling. PLoS Pathog..

[29] Aronson M.N., Meyer A.D., Györgyey J., Katul L., Vetten H.J., Gronenborn B., Timchenko T. (2000). Clink, a nanovirus-encoded protein, binds both pRB and SKP1. J. Virol..

[30] Götz S., García-Gómez J.M., Terol J., Williams T.D., Nagaraj S.H., Nueda M.J., Robles M., Talón M., Dopazo J., Conesa A. (2008). High-throughput functional annotation and data mining with the Blast2GO suite. Nucleic Acids Res..

[31] Kanehisa M., Furumichi M., Tanabe M., Sato Y., Morishima K. (2017). KEGG: New perspectives on genomes, pathways, diseases and drugs. Nucleic Acids Res..

[32] Negi S., Tak H., Bhakta S., Tiwari M., Ganapathi T.R., Singh S., Ballal A. (2025). A novel approach to enhance resistance to vascular disease by expressing cell-death-inducing fungal elicitors in the xylem tissue. Plant Biotechnol. J..

[33] Yu N.T., Feng T.C., Wang J.H., Zhang Y.L., Liu Z.X. (2011). Cloning and prokaryotic expression of *rep* gene of the Haikou isolate of Banana bunchy top virus and preparation of polyclonal antiserum against Rep. Plant Prot..

[34] Stecher G., Suleski M., Tao Q., Tamura K., Kumar S. (2026). MEGA 12.1: Cross-platform release for macOS and Linux operating systems. J. Mol. Evol..

