# Supplementary material for: BBTV Nuclear Shuttle Protein Mediates Banana Ubiquitination Pathway Dysregulation
Source: Plants (Basel). 2026 Aug 24;15(17):2571. doi: 10.3390/plants15172571 (PMC13567215; doi:10.3390/plants15172571)
Supplement: Supplementary file 1 [file plants-15-02571-s001.zip › Figure S1.pdf]

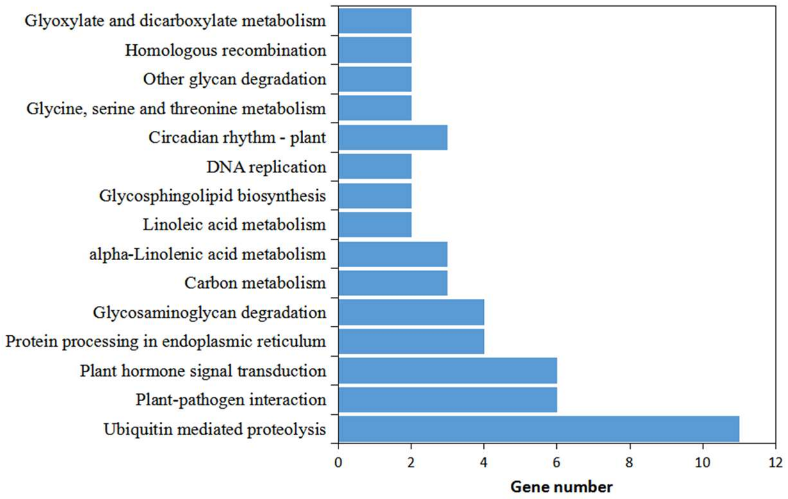

Figure S1 KEGG enrichment analysis of differentially expressed genes (DEGs) between BBTV-infected and healthy banana leaves. Bar plot displaying significantly enriched KEGG pathways. The X-axis represents the number of DEGs annotated to each pathway, and the Y-axis lists the pathway names.
