# Supplementary material for: BBTV Nuclear Shuttle Protein Mediates Banana Ubiquitination Pathway Dysregulation
Source: Plants (Basel). 2026 Aug 24;15(17):2571. doi: 10.3390/plants15172571 (PMC13567215; doi:10.3390/plants15172571)
Supplement: Supplementary file 1 [file plants-15-02571-s001.zip › Figure S2.pdf]

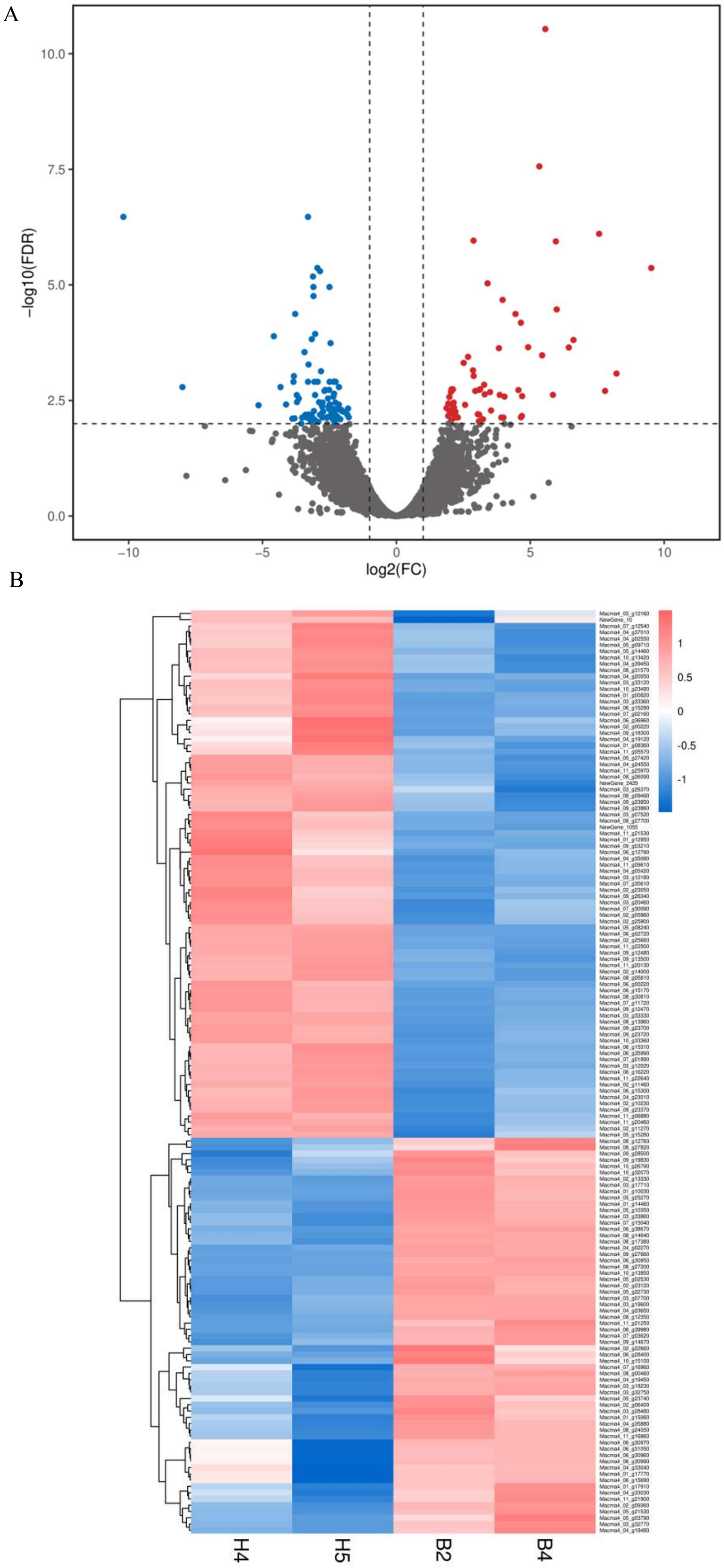

Figure S2 Transcriptomic profiling reveals differentially expressed genes (DEGs) between BBTV-infected and healthy banana leaves. The transcriptomic data used for profiling were obtained from Yu et al. (2018). (**A**) The volcano plot shows the relationship between the $\log_2$(fold

change) (x-axis) and -$\log_{10}$(FDR) (y-axis). DEGs were identified using thresholds of $|\log_2(FC)| \geq 1.7$ and a -$\log_{10}$(FDR) ≥ 2.0. (**B**) Hierarchical cluster of DEGs based on $\log_{10}$(FPKM+0.000001) expression values.
