# Supplementary material for: BBTV Nuclear Shuttle Protein Mediates Banana Ubiquitination Pathway Dysregulation
Source: Plants (Basel). 2026 Aug 24;15(17):2571. doi: 10.3390/plants15172571 (PMC13567215; doi:10.3390/plants15172571)
Supplement: Supplementary file 1 [file plants-15-02571-s001.zip › Table S1.pdf]

Table S1 List of the differently expressed genes between BBTV infected and healthy banana leaves

Gene_ID	FDR	log_2FC	Regulate	NR_annotation
Macma4_08_g27200	4.30E-06	9.52	up	hypothetical protein BHE74_00012745 [Ensete ventricosum]
Macma4_09_g27660	8.26E-04	8.22	up	hypothetical protein C4D60_Mb09t21860 [Musa balbisiana]
Macma4_10_g13950	1.97E-03	7.79	up	PREDICTED: CDT1-like protein a, chloroplastic [Musa acuminata subsp. malaccensis]
**Macma4_06_g30970**	7.84E-07	7.57	up	PREDICTED: E3 ubiquitin-protein ligase SIS3-like [Musa acuminata subsp. malaccensis]
**Macma4_06_g31050**	1.55E-04	6.61	up	PREDICTED: probable E3 ubiquitin-protein ligase HIP1 [Musa acuminata subsp. malaccensis]
**Macma4_06_g30960**	2.26E-04	6.44	up	PREDICTED: E3 ubiquitin-protein ligase SIS3-like [Musa acuminata subsp. malaccensis]
**Macma4_06_g30950**	3.40E-05	5.99	up	PREDICTED: putative RING-H2 finger protein ATL53 [Musa acuminata subsp. malaccensis]
Macma4_07_g16960	1.15E-06	5.95	up	PREDICTED: protein P21-like [Musa acuminata subsp. malaccensis]
**Macma4_06_g30990**	2.39E-03	5.85	up	PREDICTED: E3 ubiquitin-protein ligase SIS3-like [Musa acuminata subsp. malaccensis]
Macma4_11_g21250	2.93E-11	5.56	up	PREDICTED: glycine-rich cell wall structural protein 2-like [Musa acuminata subsp. malaccensis]
Macma4_06_g15690	3.34E-04	5.44	up	PREDICTED: DNA replication licensing factor MCM6 [Musa acuminata subsp. malaccensis]
Macma4_04_g19450	2.73E-08	5.34	up	PREDICTED: germin-like protein 3-8 [Musa acuminata subsp. malaccensis]
Macma4_08_g27820	2.23E-04	4.92	up	PREDICTED: phospholipase A1-II 5-like [Musa acuminata subsp. malaccensis]
Macma4_05_g23740	2.55E-03	4.69	up	PREDICTED: MDIS1-interacting receptor like kinase 2-like isoform X1 [Musa acuminata subsp. malaccensis]
Macma4_01_g17770	6.84E-03	4.68	up	PREDICTED: DNA replication licensing factor MCM4 [Musa acuminata subsp. malaccensis]
Macma4_11_g16860	6.58E-05	4.65	up	PREDICTED: protein DETOXIFICATION 49-like [Musa acuminata subsp. malaccensis]
Macma4_04_g33040	7.28E-03	4.65	up	hypothetical protein C4D60_Mb04t31640 [Musa balbisiana]
Macma4_01_g14460	1.88E-03	4.56	up	PREDICTED: dof zinc finger protein DOF1.7-like [Musa acuminata subsp. malaccensis]
Macma4_08_g12760	4.25E-05	4.45	up	hypothetical protein BHM03_00046332 [Ensete ventricosum]
Macma4_09_g28500	2.61E-03	4.04	up	PREDICTED: glycine-rich cell wall structural protein-like [Musa acuminata subsp. malaccensis]
Macma4_03_g32770	7.40E-03	4.00	up	PREDICTED: uncharacterized protein LOC103979868 [Musa acuminata subsp. malaccensis]
Macma4_04_g19480	2.11E-05	3.97	up	PREDICTED: germin-like protein 3-8 [Musa acuminata subsp. malaccensis]
Macma4_04_g33030	7.39E-03	3.92	up	PREDICTED: uncharacterized protein LOC103982826 [Musa acuminata subsp. malaccensis]
Macma4_11_g21900	2.40E-03	3.86	up	hypothetical protein C4D60_Mb11t20190 [Musa balbisiana]
Macma4_04_g35880	2.34E-04	3.83	up	PREDICTED: auxin-responsive protein SAUR36-like [Musa acuminata subsp. malaccensis]
Macma4_05_g03790	5.19E-03	3.53	up	PREDICTED: phenylalanine ammonia-lyase [Musa acuminata subsp. malaccensis]
Macma4_02_g22660	2.08E-03	3.50	up	PREDICTED: 18.1 kDa class I heat shock protein-like [Musa acuminata subsp. malaccensis]

**Gene_ID**	**FDR**	**log_2FC**	**Regulate**	**NR_annotation**
Macma4_08_g17380	9.25E-06	3.40	up	PREDICTED: probable CCR4-associated factor 1 homolog 11 [Musa acuminata subsp. malaccensis]
Macma4_01_g17910	2.33E-03	3.30	up	PREDICTED: somatic embryogenesis receptor kinase 2 [Musa acuminata subsp. malaccensis]
Macma4_01_g15060	1.44E-03	3.28	up	PREDICTED: protein SAR DEFICIENT 1-like [Musa acuminata subsp. malaccensis]
Macma4_08_g00460	8.05E-03	3.23	up	PREDICTED: retinoblastoma-related protein [Musa acuminata subsp. malaccensis]
Macma4_05_g21530	1.81E-03	3.13	up	hypothetical protein B296_00051459 [Ensete ventricosum]
Macma4_06_g38670	1.87E-03	3.09	up	PREDICTED: lectin [Musa acuminata subsp. malaccensis]
Macma4_09_g19830	6.41E-03	3.09	up	PREDICTED: probable leucine-rich repeat receptor-like protein kinase At1g35710 [Musa acuminata subsp. malaccensis]
Macma4_08_g12350	8.91E-03	3.08	up	hypothetical protein C4D60_Mb08t11930 [Musa balbisiana]
Macma4_03_g18230	6.25E-03	3.04	up	PREDICTED: heat stress transcription factor A-4b-like isoform X1 [Musa acuminata subsp. malaccensis]
Macma4_03_g07700	1.97E-03	2.94	up	hypothetical protein BHE74_00056654, partial [Ensete ventricosum]
Macma4_03_g32750	9.32E-04	2.89	up	hypothetical protein B296_00014060 [Ensete ventricosum]
Macma4_02_g23120	1.10E-06	2.88	up	PREDICTED: heat shock 70 kDa protein 14-like [Musa acuminata subsp. malaccensis]
Macma4_05_g02530	7.05E-04	2.86	up	PREDICTED: 9-cis-epoxycarotenoid dioxygenase, chloroplastic-like [Musa acuminata subsp. malaccensis]
Macma4_02_g13330	3.60E-04	2.68	up	PREDICTED: dof zinc finger protein DOF4.6-like [Musa acuminata subsp. malaccensis]
Macma4_07_g03620	3.92E-03	2.57	up	PREDICTED: probable transcription factor KAN4 [Musa acuminata subsp. malaccensis]
Macma4_07_g15040	4.86E-04	2.51	up	hypothetical protein EE612_016680 [Oryza sativa]
Macma4_02_g06400	7.39E-03	2.29	up	PREDICTED: G-type lectin S-receptor-like serine/threonine-protein kinase At2g19130 [Musa acuminata subsp. malaccensis]
Macma4_05_g25270	7.18E-03	2.27	up	PREDICTED: ribosomal RNA processing protein 1 homolog [Musa acuminata subsp. malaccensis]
Macma4_10_g15100	7.39E-03	2.24	up	PREDICTED: 22.0 kDa class IV heat shock protein-like [Musa acuminata subsp. malaccensis]
**Macma4_05_g10350**	5.49E-03	2.20	up	PREDICTED: F-box protein At2g02240 [Musa acuminata subsp. malaccensis]
Macma4_08_g24050	7.39E-03	2.18	up	PREDICTED: putative lipoxygenase 5 [Musa acuminata subsp. malaccensis]
Macma4_02_g09360	6.28E-03	2.15	up	PREDICTED: probable inactive receptor kinase At1g27190 [Musa acuminata subsp. malaccensis]
Macma4_06_g28400	4.51E-03	2.15	up	PREDICTED: NAC domain-containing protein 2-like [Musa acuminata subsp. malaccensis]
**Macma4_01_g10030**	3.51E-03	2.14	up	PREDICTED: E3 ubiquitin-protein ligase PUB23-like [Musa acuminata subsp. malaccensis]
Macma4_08_g14640	1.82E-03	2.11	up	PREDICTED: heat shock protein 83 [Musa acuminata subsp. malaccensis]

Gene_ID	FDR	log_2FC	Regulate	NR_annotation
Macma4_03_g19600	4.79E-03	2.10	up	hypothetical protein C4D60_Mb03t18480 [Musa balbisiana]
Macma4_03_g33860	5.50E-03	2.08	up	PREDICTED: uncharacterized protein LOC103979763 [Musa acuminata subsp. malaccensis]
Macma4_10_g32070	3.86E-03	2.08	up	PREDICTED: uncharacterized protein LOC103969460 [Musa acuminata subsp. malaccensis]
Macma4_06_g39980	1.81E-03	2.06	up	PREDICTED: heat shock 70 kDa protein, mitochondrial-like [Musa acuminata subsp. malaccensis]
Macma4_03_g28480	8.62E-03	2.05	up	PREDICTED: phytosulfokines-like [Musa acuminata subsp. malaccensis]
Macma4_03_g17710	2.05E-03	2.04	up	PREDICTED: dnaJ homolog subfamily B member 4-like isoform X1 [Musa acuminata subsp. malaccensis]
**Macma4_04_g02270**	2.65E-03	1.98	up	PREDICTED: probable E3 ubiquitin-protein ligase XBOS32 isoform X1 [Musa acuminata subsp. malaccensis]
Macma4_10_g26790	5.21E-03	1.97	up	PREDICTED: heat shock cognate 70 kDa protein-like [Musa acuminata subsp. malaccensis]
Macma4_09_g14670	6.80E-03	1.94	up	PREDICTED: IAA-amino acid hydrolase ILR1-like 1 [Musa acuminata subsp. malaccensis]
Macma4_04_g23650	3.68E-03	1.94	up	PREDICTED: uncharacterized protein LOC103991201 [Musa acuminata subsp. malaccensis]
Macma4_05_g22730	4.62E-03	1.87	up	hypothetical protein GW17_00036902 [Ensete ventricosum]
Macma4_09_g13500	7.28E-03	-1.78	down	PREDICTED: plasma membrane ATPase-like [Musa acuminata subsp. malaccensis]
Macma4_11_g20130	4.72E-03	-1.81	down	PREDICTED: transcription factor bHLH149-like [Musa acuminata subsp. malaccensis]
Macma4_08_g13960	6.36E-03	-1.84	down	PREDICTED: 4-coumarate--CoA ligase-like 4 [Musa acuminata subsp. malaccensis]
Macma4_11_g22640	5.49E-03	-1.99	down	PREDICTED: 28 kDa ribonucleoprotein, chloroplastic [Musa acuminata subsp. malaccensis]
Macma4_08_g09490	5.21E-03	-2.06	down	PREDICTED: probable glycerol-3-phosphate dehydrogenase [NAD(+)] 1, cytosolic [Musa acuminata subsp. malaccensis]
Macma4_09_g12480	5.02E-03	-2.06	down	PREDICTED: equilibrative nucleotide transporter 3 isoform X1 [Musa acuminata subsp. malaccensis]
**Macma4_10_g23490**	8.04E-03	-2.09	down	PREDICTED: probable BOI-related E3 ubiquitin-protein ligase 3 isoform X2 [Musa acuminata subsp. malaccensis]
Macma4_09_g23850	3.86E-03	-2.15	down	PREDICTED: uncharacterized protein LOC103998646 [Musa acuminata subsp. malaccensis]
Macma4_05_g14460	1.62E-03	-2.15	down	PREDICTED: fatty acid desaturase DES3 [Musa acuminata subsp. malaccensis]
Macma4_03_g33120	4.93E-03	-2.21	down	PREDICTED: heparanase-like protein 2 [Musa acuminata subsp. malaccensis]
Macma4_05_g09710	6.95E-03	-2.22	down	PREDICTED: ELF3-like protein 2 [Musa acuminata subsp. malaccensis]
Macma4_04_g23010	7.35E-03	-2.26	down	PREDICTED: pentatricopeptide repeat-containing protein At2g44880-like [Musa acuminata subsp. malaccensis]
Macma4_03_g12020	1.25E-03	-2.27	down	PREDICTED: putative pentatricopeptide repeat-containing protein At3g13770, mitochondrial [Musa

Gene_ID	FDR	log_2FC	Regulate	NR_annotation
				acuminata subsp. malaccensis]
Macma4_01_g00820	3.51E-03	-2.28	down	PREDICTED: photosynthetic NDH subunit of subcomplex B 1, chloroplastic [Musa acuminata subsp. malaccensis]
Macma4_07_g01890	8.04E-03	-2.29	down	PREDICTED: uncharacterized protein LOC103990890 [Musa acuminata subsp. malaccensis]
Macma4_09_g26340	6.36E-03	-2.30	down	PREDICTED: NADP-dependent D-sorbitol-6-phosphate dehydrogenase [Musa acuminata subsp. malaccensis]
Macma4_06_g15300	9.62E-03	-2.30	down	PREDICTED: uncharacterized protein LOC103988384 [Musa acuminata subsp. malaccensis]
**Macma4_05_g27420**	2.25E-03	-2.33	down	PREDICTED: E3 ubiquitin-protein ligase RHA1B-like [Musa acuminata subsp. malaccensis]
Macma4_06_g16220	1.25E-03	-2.33	down	PREDICTED: heavy metal-associated isoprenylated plant protein 22-like [Musa acuminata subsp. malaccensis]
Macma4_10_g33360	1.23E-03	-2.33	down	PREDICTED: myb-related protein Hv1 [Musa acuminata subsp. malaccensis]
Macma4_08_g31570	4.81E-03	-2.35	down	PREDICTED: filament-like plant protein 3 isoform X1 [Musa acuminata subsp. malaccensis]
NewGene_1055	2.61E-03	-2.36	down	hypothetical protein GW17_00037253, partial [Ensete ventricosum]
Macma4_02_g05960	8.09E-03	-2.38	down	PREDICTED: polygalacturonase QRT3 [Musa acuminata subsp. malaccensis]
Macma4_07_g30090	6.28E-03	-2.44	down	PREDICTED: uncharacterized protein LOC103993105 [Musa acuminata subsp. malaccensis]
Macma4_11_g25970	1.82E-04	-2.46	down	PREDICTED: uncharacterized protein LOC103972691 [Musa acuminata subsp. malaccensis]
Macma4_03_g12180	3.86E-03	-2.49	down	PREDICTED: BTB/POZ domain-containing protein At5g47800-like [Musa acuminata subsp. malaccensis]
Macma4_11_g22500	1.11E-05	-2.50	down	PREDICTED: uncharacterized protein LOC103972189 [Musa acuminata subsp. malaccensis]
Macma4_09_g12470	1.25E-03	-2.50	down	PREDICTED: disease resistance RPP13-like protein 4 [Musa acuminata subsp. malaccensis]
Macma4_03_g33330	1.87E-03	-2.51	down	PREDICTED: glucan endo-1,3-beta-glucosidase 7-like [Musa acuminata subsp. malaccensis]
Macma4_01_g08360	4.81E-03	-2.52	down	PREDICTED: protein QUIRKY-like [Musa acuminata subsp. malaccensis]
Macma4_01_g12950	4.21E-03	-2.53	down	PREDICTED: transcription factor bHLH76-like [Musa acuminata subsp. malaccensis]
Macma4_02_g14000	2.86E-03	-2.56	down	PREDICTED: uncharacterized protein At1g65710 [Musa acuminata subsp. malaccensis]
Macma4_05_g15280	7.28E-03	-2.59	down	PREDICTED: uncharacterized protein LOC108952821 [Musa acuminata subsp. malaccensis]
Macma4_04_g35080	7.39E-03	-2.59	down	PREDICTED: tubulin beta-7 chain-like [Musa acuminata subsp. malaccensis]
Macma4_06_g00220	7.26E-03	-2.60	down	hypothetical protein B296_00008635 [Ensete ventricosum]
Macma4_07_g12540	1.87E-03	-2.65	down	PREDICTED: zinc finger protein CONSTANS-LIKE 6-like isoform X1 [Musa acuminata subsp. malaccensis]

Gene_ID	FDR	log_2FC	Regulate	NR_annotation
Macma4_08_g26090	1.97E-03	-2.69	down	PREDICTED: transcription factor bHLH49-like [Musa acuminata subsp. malaccensis]
Macma4_07_g11720	5.50E-03	-2.74	down	PREDICTED: homeobox-leucine zipper protein HOX13-like [Musa acuminata subsp. malaccensis]
Macma4_02_g11270	7.39E-03	-2.75	down	PREDICTED: beta-hexosaminidase 2 [Musa acuminata subsp. malaccensis]
Macma4_09_g03210	3.51E-03	-2.76	down	PREDICTED: metal tolerance protein 5-like [Musa acuminata subsp. malaccensis]
Macma4_08_g05810	4.35E-03	-2.77	down	PREDICTED: 50S ribosomal protein L3-1, chloroplastic-like [Musa acuminata subsp. malaccensis]
Macma4_03_g26370	3.86E-03	-2.80	down	PREDICTED: glycine cleavage system H protein 2, mitochondrial [Musa acuminata subsp. malaccensis]
Macma4_09_g23860	7.37E-04	-2.82	down	PREDICTED: uncharacterized protein LOC103998647 [Musa acuminata subsp. malaccensis]
Macma4_03_g07520	6.43E-03	-2.85	down	PREDICTED: probable auxin efflux carrier component 1c [Musa acuminata subsp. malaccensis]
Macma4_06_g15170	5.04E-06	-2.85	down	PREDICTED: uncharacterized protein LOC103987727 [Musa acuminata subsp. malaccensis]
Macma4_10_g13420	3.44E-03	-2.89	down	hypothetical protein C4D60_Mb10t19980 [Musa balbisiana]
Macma4_02_g10230	9.18E-03	-2.92	down	PREDICTED: uncharacterized protein LOC108952141 [Musa acuminata subsp. malaccensis]
Macma4_11_g20460	1.25E-03	-2.94	down	PREDICTED: sphingolipid delta(4)-desaturase DES1-like [Musa acuminata subsp. malaccensis]
Macma4_06_g35990	4.30E-06	-2.95	down	PREDICTED: uncharacterized protein LOC103989693 [Musa acuminata subsp. malaccensis]
Macma4_09_g18300	2.41E-03	-3.04	down	PREDICTED: protein EARLY RESPONSIVE TO DEHYDRATION 15-like [Musa acuminata subsp. malaccensis]
Macma4_04_g05420	1.15E-04	-3.04	down	PREDICTED: B-box zinc finger protein 20-like [Musa acuminata subsp. malaccensis]
Macma4_03_g33360	8.13E-03	-3.06	down	PREDICTED: scopoletin glucosyltransferase-like [Musa acuminata subsp. malaccensis]
Macma4_11_g21530	1.25E-03	-3.06	down	hypothetical protein C4D60_Mb11t19840 [Musa balbisiana]
Macma4_04_g02550	5.34E-03	-3.08	down	PREDICTED: xylogen-like protein 11 [Musa acuminata subsp. malaccensis]
Macma4_09_g23700	1.11E-05	-3.10	down	PREDICTED: clavaminate synthase-like protein At3g21360 [Musa acuminata subsp. malaccensis]
Macma4_09_g23720	1.74E-05	-3.10	down	PREDICTED: clavaminate synthase-like protein At3g21360 [Musa acuminata subsp. malaccensis]
Macma4_06_g15290	6.58E-06	-3.12	down	PREDICTED: uncharacterized protein LOC103987739 [Musa acuminata subsp. malaccensis]
Macma4_11_g05570	1.49E-04	-3.16	down	PREDICTED: protein FAM179B-like [Musa acuminata subsp. malaccensis]
Macma4_03_g20460	6.80E-03	-3.17	down	PREDICTED: blue copper protein-like [Musa acuminata subsp. malaccensis]
**Macma4_07_g30610**	8.68E-03	-3.20	down	PREDICTED: F-box protein At1g55000-like [Musa acuminata subsp. malaccensis]
Macma4_04_g19120	6.36E-03	-3.27	down	PREDICTED: ABC transporter B family member 2-like [Musa acuminata subsp. malaccensis]
Macma4_04_g39450	5.28E-04	-3.28	down	PREDICTED: zinc finger protein CONSTANS-LIKE 16-like [Musa acuminata subsp. malaccensis]
Macma4_09_g23370	1.25E-03	-3.30	down	PREDICTED: probable NAD(P)H dehydrogenase subunit CRR3, chloroplastic [Musa acuminata

Gene_ID	FDR	log_2FC	Regulate	NR_annotation
				subsp. malaccensis]
Macma4_04_g24550	3.38E-07	-3.30	down	PREDICTED: pentatricopeptide repeat-containing protein At2g02980, chloroplastic [Musa acuminata subsp. malaccensis]
Macma4_08_g30810	7.39E-03	-3.35	down	PREDICTED: leucine-rich repeat receptor-like serine/threonine-protein kinase BAM1 [Musa acuminata subsp. malaccensis]
Macma4_11_g06880	6.43E-03	-3.40	down	PREDICTED: protein-lysine methyltransferase METTL21D-like [Musa acuminata subsp. malaccensis]
Macma4_04_g20050	2.85E-04	-3.43	down	PREDICTED: protein WVD2-like 1 isoform X1 [Musa acuminata subsp. malaccensis]
Macma4_06_g12790	7.40E-03	-3.52	down	PREDICTED: cyclin-A3-1-like [Musa acuminata subsp. malaccensis]
Macma4_11_g09610	9.98E-03	-3.55	down	PREDICTED: probable receptor-like protein kinase At5g61350 [Musa acuminata subsp. malaccensis]
Macma4_04_g37010	2.86E-03	-3.65	down	ig1-as2 like1, partial [Zea mays]
Macma4_02_g11460	3.43E-03	-3.71	down	PREDICTED: probable polygalacturonase [Musa acuminata subsp. malaccensis]
Macma4_02_g00220	2.41E-03	-3.72	down	PREDICTED: fatty-acid-binding protein 1-like [Musa acuminata subsp. malaccensis]
Macma4_02_g25900	4.25E-05	-3.78	down	PREDICTED: UDP-glucuronate:xylan alpha-glucuronosyltransferase 2-like isoform X1 [Musa acuminata subsp. malaccensis]
Macma4_08_g27700	7.68E-03	-3.80	down	PREDICTED: leucine-rich repeat extensin-like protein 4 [Musa acuminata subsp. malaccensis]
Macma4_07_g02160	9.39E-04	-3.83	down	PREDICTED: probable tyrosine-protein phosphatase At1g05000 [Musa acuminata subsp. malaccensis]
Macma4_02_g23050	7.82E-03	-3.88	down	PREDICTED: probable LRR receptor-like serine/threonine-protein kinase At3g47570 [Musa acuminata subsp. malaccensis]
Macma4_06_g36960	3.86E-03	-4.12	down	PREDICTED: uncharacterized protein LOC103974427 [Musa acuminata subsp. malaccensis]
Macma4_03_g12160	1.62E-03	-4.33	down	PREDICTED: fructose-bisphosphate aldolase 5, cytosolic [Musa acuminata subsp. malaccensis]
Macma4_02_g25660	1.29E-04	-4.58	down	PREDICTED: protein UPSTREAM OF FLC-like [Musa acuminata subsp. malaccensis]
Macma4_06_g02720	1.62E-03	-7.99	down	PREDICTED: beta-galactosidase-like [Musa acuminata subsp. malaccensis]
Macma4_05_g08240	3.38E-07	-10.20	down	PREDICTED: alpha-amylase isozyme 3C-like [Musa acuminata subsp. malaccensis]
NewGene_10	4.01E-03	-5.15	down	—
NewGene_1055	2.61E-03	-2.36	down	—
NewGene_2429	1.25E-03	-3.85	down	—
